# Supplementary material for: Layered roles of fruitless isoforms in specification and function of male aggression-promoting neurons in Drosophila
Source: eLife. 2020 Apr 21;9:e52702. doi: 10.7554/eLife.52702 (PMC7173971; doi:10.7554/eLife.52702)
Supplement: Supplementary file 1. [file elife-52702-supp1.docx]

**Supplementary File 1**

| **Key Resources Table** | | | | |
| --- | --- | --- | --- | --- |
| **Reagent type (species) or resource** | **Designation** | **Source or reference** | **Identifiers** | **Additional information** |
| gene (*Drosophila melanogaster*) | *fru* | FlyBase | FLYB:FBgn0004652 |  |
| strain, strain background (*Drosophila melanogaster*) | Canton-S | Hoyer et al., 2008 (DOI:10.1016/j.cub.2007.12.052) |  | Gift from David Anderson (California Institute of Technology) |
| strain, strain background (*Drosophila melanogaster*) | Canton-S (isogenic) | von Philipsborn et al., 2014 (DOI:10.1016/j.cub.2013.12.015) |  | Gift from Anne von Philipsborn (Aarhus Univ.) |
| strain, strain background (*Drosophila melanogaster*) | *Tk-GAL4^1^* | Asahina et al., 2014 (DOI:10.1016/j.cell.2013.11.045) | BDSC:51975; FLYB:FBal0288518; RRID:BDSC_51975 |  |
| strain, strain background (*Drosophila melanogaster*) | *Otd-nls:FLPo* (in attP40) | Asahina et al., 2014 (DOI:10.1016/j.cell.2013.11.045) | FLYB: FBtp0093566 |  |
| genetic reagent (*Drosophila melanogaster*) | *NP2631* | Yu et al., 2010 (DOI:10.1016/j.cub.2010.08.025) | DGGR:104266; FLYB:FBti0034959; RRID:DGGR_104266 | Gift from Daisuke Yamamoto (Tohoku Univ.) |
| genetic reagent (*Drosophila melanogaster*) | *20XUAS>myr:TopHAT2>CsChrimson:tdTomato* (in VK00022) | Rubin lab (HHMI Janelia Research Campus) |  | Gift from David Anderson (California Institute of Technology) |
| genetic reagent (*Drosophila melanogaster*) | *20XUAS>myr:TopHAT2>CsChrimson:tdTomato* (in VK00005) | Duistermars et al., 2018 (DOI:10.1016/j.neuron.2018.10.027) |  | Gift from David Anderson (California Institute of Technology) |
| genetic reagent (*Drosophila melanogaster*) | *20XUAS>myr:TopHAT2>CsChrimson:tdTomato* (in attP2) | Rubin lab (HHMI Janelia Research Campus) |  | Gift from David Anderson (California Institute of Technology) |
| genetic reagent (*Drosophila melanogaster*) | *20XUAS-IVS-Syn21-GCaMP6f* (codon-optimized)*-p10* (in su(Hw)attP5) | Rubin lab (HHMI Janelia Research Campus) |  | Gift from David Anderson (California Institute of Technology) |
| genetic reagent (*Drosophila melanogaster*) | *13XlexAop2-IVS-Syn21-GCaMP6f* (codon-optimized)*-p10* (in su(Hw)attP5) | Rubin lab (HHMI Janelia Research Campus) |  | Gift from David Anderson (California Institute of Technology) |
| genetic reagent (*Drosophila melanogaster*) | *fru^M^* | Demir & Dickson, 2005 (DOI:10.1016/j.cell.2005.04.027) | BDSC:66874; FLYB:FBal0179807; RRID:BDSC_66874 | Gift from Barry Dickson (HHMI Janelia Research Campus) |
| genetic reagent (*Drosophila melanogaster*) | *fru^F^* | Demir & Dickson, 2005 (DOI:10.1016/j.cell.2005.04.027) | BDSC:66873; FLYB:FBal0191136; RRID:BDSC_66873 | Gift from Barry Dickson (HHMI Janelia Research Campus) |
| genetic reagent (*Drosophila melanogaster*) | *fru^FLP^* | Yu et al., 2010 (DOI:10.1016/j.cub.2010.08.025) | BDSC:66870; FLYB:FBal0248671; RRID:BDSC_66870 | Gift from Barry Dickson (HHMI Janelia Research Campus) |
| genetic reagent (*Drosophila melanogaster*) | *dsx^FLP^* | Rezaval et al., 2014 (DOI:10.1016/j.cub.2013.12.051) | FLYB:FBal0296301 | Gift from Stephen Goodwin (Univ. Oxford) |
| genetic reagent (*Drosophila melanogaster*) | *fru^Δ A^* | Neville et al., 2014 (DOI:10.1016/j.cub.2013.11.035) | FLYB:FBal0295056 | Gift from Stephen Goodwin (Univ. Oxford) |
| genetic reagent (*Drosophila melanogaster*) | *fru^Δ B^* | Neville et al., 2014 (DOI:10.1016/j.cub.2013.11.035) | FLYB:FBal0295057 | Gift from Stephen Goodwin (Univ. Oxford) |
| genetic reagent (*Drosophila melanogaster*) | *fru^Δ C^* | Billeter et al., 2006 (DOI:10.1016/j.cub.2006.04.039) | FLYB:FBal0245249 | Gift from Stephen Goodwin (Univ. Oxford) |
| genetic reagent (*Drosophila melanogaster*) | *fru^P1.LexA^* | Mellert et al., 2010 (DOI: 10.1242/dev.045047) | BDSC:66698; FLYB:FBal0244777; RRID:BDSC_66698 | Gift from Bruce Baker (HHMI Janelia Research Campus) |
| genetic reagent (*Drosophila melanogaster*) | *fru^B1^* | von Philipsborn et al., 2014 (DOI:10.1016/j.cub.2013.12.015) | FLYB:FBal0296342 | Gift from Anne von Philipsborn (Aarhus Univ.) |
| genetic reagent (*Drosophila melanogaster*) | *fru^B2^* | von Philipsborn et al., 2014 (DOI:10.1016/j.cub.2013.12.015) | FLYB:FBal0296343 | Gift from Anne von Philipsborn (Aarhus Univ.) |
| genetic reagent (*Drosophila melanogaster*) | *fru^4-40^* | Bloomington *Drosophila* Resource Center | BDRC:66692; FLYB:FBal0126397; RRID:BDSC_66692 |  |
| genetic reagent (*Drosophila melanogaster*) | *R71G01-LexA* (in attP2) | The accompanying paper |  | Created by targeting the GMR71G01-LexA plasmid (gift from Rubin lab) into the attP2 landing site. See Materials and Methods: Fly Strains of the accompanying paper for details. |
| antibody | anti-DsRed (Rabbit polyclonal) | Clontech | Cat # 632496; RRID: AB_10013483 | IHC (1:1,000) |
| antibody | anti-BRP (Mouse monoclonal nc82) | Developmental Studies Hybridoma Bank | nc82 (concentrate); RRID: AB_2314866 | IHC (1:100) |
| antibody | anti-GFP (Chicken polyclonal) | Abcam | Cat # ab13970; RRID:AB_300798 | IHC (1:1,000) |
| antibody | anti-FruM (Rabbit polyclonal) | Stockinger et al., 2005 (DOI:10.1016/j.cell.2005.04.026) |  | Gift from Barry Dickson (HHMI Janelia Research Campus). IHC (1:10,000) |
| antibody | anti-FruM (Guinea pig polyclonal) | The accompanying paper |  | Generated by Michael Perry (UC San Diego) and provided to the authors. This statement is duplicated from the Key Resources Table of the accompanying paper. IHC (1:100) |
| antibody | anti-DsxM (Rat polyclonal) | The accompanying paper |  | Generated by Michael Perry (UC San Diego) and provided to the authors. This statement is duplicated from the Key Resources Table of the accompanying paper. IHC (1:100) |
| antibody | anti-HA (Rat polyclonal) | Roche | Cat # 11867423001; RRID:AB_390918 | IHC (1:100) |
| antibody | anti-chicken IgY Alexa 488 (Goat polyclonal) | ThermoFisher Scientific | Cat # A11039; RRID:AB_2534096 | IHC (1:100) |
| antibody | anti-rat IgG Alexa 488 (Goat polyclonal) | ThermoFisher Scientific | Cat # A11006; RRID:AB_2534074 | IHC (1:100) |
| antibody | anti-rabbit IgG Alexa 568 (Goat polyclonal) | ThermoFisher Scientific | Cat # A11036; RRID:AB_10563566 | IHC (1:100) |
| antibody | anti-mouse IgG Alexa 633 (Goat polyclonal) | ThermoFisher Scientific | Cat # A21052; RRID:AB_2535719 | IHC (1:100) |
| antibody | anti-guinea pig IgG Alexa 633 (Goat polyclonal) | ThermoFisher Scientific | Cat # A21105; RRID:AB_2535757 | IHC (1:100) |
| chemical compound, drug | Vectashield | Vector Laboratories | Cat # H-1000; RRID:AB_2336789 |  |
| chemical compound, drug | FocusClear | CelExplorer Labs, Taiwan | Cat # FC-101 |  |
| chemical compound, drug | MountClear | CelExplorer Labs, Taiwan | Cat # MC-301 |  |
| chemical compound, drug | all-*trans* retinal | MilliporeSigma | Cat # R2500 | Used at 0.2mM |
| software, algorithm | Fiji | Schindelin et al., 2012 (DOI:10.1038/nmeth.2019) | RRID:SCR_002285 | https://fiji.sc/ |
| software, algorithm | Computational Morphometry Tookit (CMTK) | Rohlfing & Maurer, 2003 (DOI:10.1109/titb.2003.808506) | RRID:SCR_002234 | https://www.nitrc.org/projects/cmtk |
| software, algorithm | Fiji plugin for CMTK | Jefferis et al., 2007 (DOI:10.1016/j.cell.2007.01.040) |  | https://github.com/jefferis/fiji-cmtk-gui |
| software, algorithm | FluoRender | Wan, Otsuna, Chien, & Hansen, 2009 (DOI:10.1109/TVCG.2009.118) | RRID:SCR_014303 | https://github.com/SCIInstitute/fluorender |
| software, algorithm | MATLAB | The Mathworks, Inc. | RRID:SCR_001622 |  |
| software, algorithm | BIAS | IORodeo |  | https://bitbucket.org/iorodeo/bias |
| software, algorithm | FlyTracker | Eyjolfsdottir et al., 2014 (DOI:https://doi.org/10.1007/978-3-319-10605-2_50) |  | http://www.vision.caltech.edu/Tools/FlyTracker/ |
| software, algorithm | JAABA | Kabra et al., 2013 (DOI: 10.1038/nmeth.2281) |  | https://sourceforge.net/projects/jaaba/files/ |
| software, algorithm | Olympus Viewer Plugin for ImageJ | Olympus Corporation |  | https://imagej.net/OlympusImageJPlugin |
| other | Multiphoton laser scanning microscope | Olympus Corporation | FV-MPE-RS |  |
| other | Tunable ultrafast laser | Newport Corporation | Spectra-Physics Insight DL Dual-OL |  |
