# Supplementary material for: Layered roles of fruitless isoforms in specification and function of male aggression-promoting neurons in Drosophila
Source: eLife. 2020 Apr 21;9:e52702. doi: 10.7554/eLife.52702 (PMC7173971; doi:10.7554/eLife.52702)
Supplement: Transparent reporting form [file elife-52702-transrepform.docx]

***eLife’s* transparent reporting form**

We encourage authors to provide detailed information *within their submission* to facilitate the interpretation and replication of experiments. Authors can upload supporting documentation to indicate the use of appropriate reporting guidelines for health-related research (see [EQUATOR Network](http://www.equator-network.org/%20)), life science research (see the [BioSharing Information Resource](https://biosharing.org/)), or the [ARRIVE guidelines](http://www.plosbiology.org/article/info:doi/10.1371/journal.pbio.1000412) for reporting work involving animal research. Where applicable, authors should refer to any relevant reporting standards documents in this form.

If you have any questions, please consult our Journal Policies and/or contact us: [editorial@elifesciences.org](mailto:editorial@elifesciences.org).

**Sample-size estimation**

- You should state whether an appropriate sample size was computed when the study was being designed
- You should state the statistical method of sample size computation and any required assumptions
- If no explicit power analysis was used, you should describe how you decided what sample (replicate) size (number) to use

See Materials and Methods for power analyses on behavioural and histological data. Please note that we do not assume a normal distribution for behavioral data and neurite volume data, which makes a model-based power analysis with a standard method not feasible. Statistical analyses for these data are non-parametric.

Please outline where this information can be found within the submission (e.g., sections or figure legends), or explain why this information doesn’t apply to your submission:

**Replicates**

- You should report how often each experiment was performed
- You should include a definition of biological versus technical replication
- The data obtained should be provided and sufficient information should be provided to indicate the number of independent biological and/or technical replicates
- If you encountered any outliers, you should describe how these were handled
- Criteria for exclusion/inclusion of data should be clearly stated
- High-throughput sequence data should be uploaded before submission, with a private link for reviewers provided (these are available from both GEO and ArrayExpress)

Please outline where this information can be found within the submission (e.g., sections or figure legends), or explain why this information doesn’t apply to your submission:

Sample sizes are indicated in each figure panel, and replications are all biological (each sample comes from single individuals, and no animals were tested multiple times). As stated in Materials and Methods, a fly pair was removed from behavioral data analysis when (1) one of the two flies were killed during introduction to the chamber, (2) the wings of either fly was stuck at the extended position, or (3) the discrimination of the two flies was impossible due to wing damage of a tester fly. All other samples are included for data analysis.

**Statistical reporting**

- Statistical analysis methods should be described and justified
- Raw data should be presented in figures whenever informative to do so (typically when N per group is less than 10)
- For each experiment, you should identify the statistical tests used, exact values of N, definitions of center, methods of multiple test correction, and dispersion and precision measures (e.g., mean, median, SD, SEM, confidence intervals; and, for the major substantive results, a measure of effect size (e.g., Pearson's r, Cohen's d)
- Report exact p-values wherever possible alongside the summary statistics and 95% confidence intervals. These should be reported for all key questions and not only when the p-value is less than 0.05.

Please outline where this information can be found within the submission (e.g., sections or figure legends), or explain why this information doesn’t apply to your submission:

Statistical methods can be found in figure legends, and additional information can be found in Materials and Method section. Confidence intervals cannot be calculated for nonparametric datasets. Exact p values for all statistical tests (along with all data points) are indicated on source data sheets for each figure panel (below data points).

(For large datasets, or papers with a very large number of statistical tests, you may upload a single table file with tests, Ns, etc., with reference to sections in the manuscript.)

**Group allocation**

- Indicate how samples were allocated into experimental groups (in the case of clinical studies, please specify allocation to treatment method); if randomization was used, please also state if restricted randomization was applied
- Indicate if masking was used during group allocation, data collection and/or data analysis

Please outline where this information can be found within the submission (e.g., sections or figure legends), or explain why this information doesn’t apply to your submission:

Samples were allocated into experimental groups according to the genotypes, sex of the target flies, and other relevant experimental conditions (such as LED intensities for optogenetic experiments). All of these conditions are indicated in each figure panel and legend. We designed our experiments so that relevant genotypes and/or treatments were performed on the same day, in the same movie where possible, across multiple days, so as to control potential day-to-day variabilities.

**Additional data files (“source data”)**

- We encourage you to upload relevant additional data files, such as numerical data that are represented as a graph in a figure, or as a summary table
- Where provided, these should be in the most useful format, and they can be uploaded as “Source data” files linked to a main figure or table
- Include model definition files including the full list of parameters used
- Include code used for data analysis (e.g., R, MatLab)
- Avoid stating that data files are “available upon request”

Please indicate the figures or tables for which source data files have been provided:

Source immunohistological images, data points for histological and behavioral experiments (along with exact p values, as stated above) for all figures are available in the source data file or supporting files. Custom MATLAB codes are available on Github, as indicated in Materials and Methods.
